# Facial-onset sensory and motor neuronopathy with myasthenia gravis: A case report

**DOI:** 10.1097/MD.0000000000034215

**Published:** 2023-11-17

**Authors:** Chengyu Pan, Xiangrong Yang, Zhenzhen Tai, Zhiwei Zhou, Renfang Hao, Jin Wang, Tao Liang

**Affiliations:** a Department of Neurology, Affiliated Hospital of Zunyi Medical University, Zunyi, China; b Department of Ultrasound, Guizhou Aerospace Hospital, Zunyi, China.

**Keywords:** facial, myasthenia gravis, onset sensory and motor neuronopathy

## Abstract

**Rationale:**

Facial-onset sensory and motor neuronopathy (FOSMN) is a greatly rare disease, so far, autopsy evidence that is associated with neurodegenerative. Myasthenia gravis (MG) is an antibody-mediated and complement-involved acquired autoimmune disorder of the post-synaptic neuromuscular junction. There have been few reports about if there is related between the 2. In this study, we present the case of a man who was diagnosed as FOSMN with MG in continuity.

**Patient concerns:**

The patient chief complaints were right-side facial numbness and right-eyelid incomplete closure, followed by slurred speech and dysphagia, and the symptoms gradually progressed. The patient serum was positive for anti-AchR and anti-Titin antibodies.

**Diagnoses:**

The patient was diagnosed FOSMN with MG.

**Interventions:**

The patient symptoms were relieved after pyridostigmine bromide and prednisolone treatment.

**Outcomes:**

Symptoms have improved.

**Lessons:**

Facial-onset sensory and motor neuronopathy and MG have disparate clinical features. Therefore, we reported a rare case in which the 2 conditions concurrently existed. Immune dysfunction might be the pathogenesis of this association, while there is no definite evidence to support it, further studies are needed.

## 1. Introduction

Facial-onset sensory and motor neuronopathy (FOSMN) syndrome is a rare disorder, at present, the pathological mechanisms have not yet been fully elucidated. The leading hypotheses are that FOSMN syndrome is either a neurodegenerative or an autoimmune disease. Vucic et al^[1]^ reported that no inflammatory changes were found in 2 autopsied patients, suggesting that FOSMN syndrome is a neurodegenerative disease with primary invasion of sensory and motor neurons. Myasthenia gravis (MG) is an antibody-mediated autoimmune disorder that affects the postsynaptic neuromuscular junction, leading to fluctuating weakness of skeletal muscles. In terms of pathogenesis, FOSMN and MG are 2 different diseases. Whether there is a correlation between the 2 diseases has not been reported.

## 2. Case presentation

**Figure 1.**
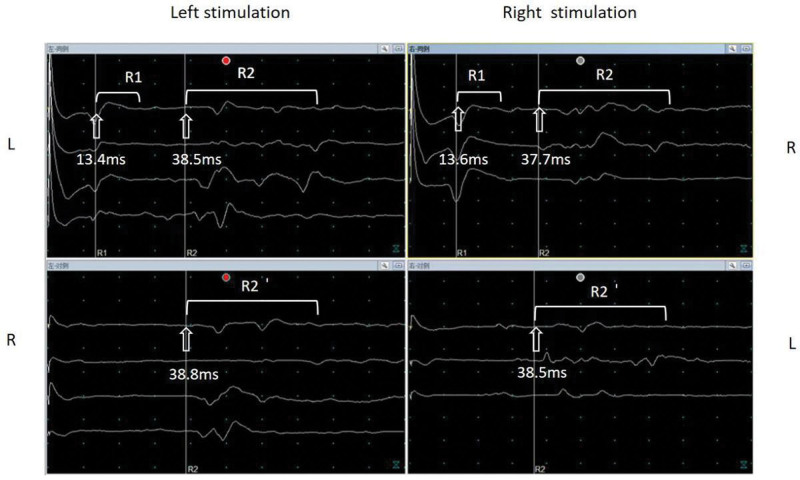
Evaluation of the blink reflex. The blink reflex shows impaired brainstem function and delayed R1, R2, and R2’ responses after ipsilateral and contralateral stimulation. The arrows indicate the delay time for each response. Normal value (ms): R1 10.0 ± 0.6, ipsilateral R2 29.3 ± 1.7, contralateral R2’ 29.2 ± 1.8.

The 41-year-old male was admitted to our hospital with progressive numbness of the right-side of the face and incomplete closure of the right eyelid for 6 years, slurred speech for 3 years, and dysphagia for 1 month. Six years ago, the patient had numbness on the right side of the face and incomplete closure of the right eyelid without obvious cause. Neurotrophic therapy was given, his medical condition was not improved, and the symptoms gradually progressed. Three years ago, his speech was slurred, he could not show facial expressions on the right side, his eyebrows were asymmetrical, and he visited our hospital many times. He received treatment to improve circulation and nourish nerves, but his symptoms did not improve. Easy to feel tired. One month before admission, after tooth extraction under local anesthesia, he experienced dysphagia and dyspnea with fluctuating symptoms, especially after the event. He found that his larynx was shifted to the right and his lower neck was swollen and uncomfortable, without dizziness, headache, muscle pain, or fasciculation. His personal and family history were unremarkable. Physical examination results were as follows: temperature, 36.5°C; and blood pressure, 120/72 mm Hg. No abnormalities were found in cardiopulmonary and abdominal examinations. In nervous system examinations, the patient was noted to be conscious, with slightly unclear speech. He answered questions correctly. The corneal reflex was absent bilaterally, the right eyebrow was lower than the left, and the masticatory muscle was weak. The nasolabial fold on the right side was slightly shallow, the muscle strength of both eyes closed and lips closed was poor, and the tympanic membranes were ruptured. The uvula was in the middle, the palatal arch was raised slightly, and the pharyngeal reflex was decreased. His tongue was skewed to the right, with tongue muscle atrophy and tremor (Supplementary Material 1, http://links.lww.com/MD/K533). Bilateral temporal and masseter muscle atrophy was noted. Weak chewing, with the jaw tilted to the left and the glottis to the right when the mouth was open, was noted. The neck was thickened. Head-up muscle strength was 5/5 according to Medical Research Council (MRC) grading, and limb muscle strength was 5/5. He showed decreased acupuncture sensation on the face, and his skin was similar to that of an onion. The patient showed slightly hyperactive tendon reflexes in limbs, although pathological signs were not elicited. Laboratory test results were as follows: routine blood, urine, and stool parameters were normal; liver and kidney function parameters were normal; and blood glucose, blood lipids, erythrocyte sedimentation rate, thyroid function parameters were normal. Tests for the anti-nuclear antibody and anti-nuclear antibody spectrum were all negative. The results of MG-related antibody tests (performed at Sheng Yuan Medical Laboratory) were as follows: anti-AchR antibody, 7.17 nmol/L (reference < 0.5 nmol/L); and anti-titin antibody, 1.55 nmol/L (reference < 1 nmol/L). The neostigmine test was positive. Chest computed tomography showed pneumonia in the lower lobe of both lungs. No obvious abnormalities were found in head magnetic resonance imaging scans. Low frequency repeated electrical stimulation test was positive. Electromyography (muscles examined included facial muscles and some limb muscles) showed no spontaneous power generation, and light contraction motor unit potential changes were not obvious. The results of a nerve conduction study were as follows: right facial nerve (temporal branch) and left facial nerve (marginal mandibular branch) motor nerve conduction velocities (MCVs) were slowed compared to their respective normal MCV ranges; sensory nerve conduction velocities sensory nerve conduction velocity of the right sural nerve were slowed, the sensory nerve action potential amplitude was decreased, the sensory nerve conduction velocity of the right ulnar nerve was slowed; and the MCVs and F waves of the right median nerve, left ulnar nerve, right tibial nerve, and common peroneal nerve were within the normal ranges. The results of a repetitive electrical stimulation study were as follows: recordings of the orbicularis oculi muscle at low- and high-frequency stimulation and compound muscle motor potential amplitude showed a decreasing trend. The results of evoked potential studies were as follows: trigeminal somatosensory evoked potentials were slightly abnormal; no obvious abnormalities were found in either upper limb somatosensory evoked potentials or visual and auditory evoked potentials. The blink reflexes of the R1, R2 and R2’ responses were delayed (Fig. 1). On the basis of the symptoms, signs, laboratory examination results and magnetic resonance imaging findings of this patient, the diagnosis of the FOSMN with MG were considered. Symptoms were improved slightly after treatment with vitamin B1 and methylcobalamin to nourish nerves, pyridostigmine bromide 60 mg for 1 to 3 times a day, prednisolone 60 mg per day (with gradual taper), potassium supplementation, calcium supplementation, etc.

Prednisone acetate 60mg was taken in the morning and maintained for 8 weeks, then gradually reduced to 5mg every 2 weeks, to 20mg every 4 weeks and 5mg every 4 weeks, to 10mg long-term oral maintenance. During the treatment, he noticed an improvement in the dysphagia, dyspnea and dysesthesia of his face. The improvement lasted for at least a period of 4 months. However, there was no progress for 20 months.

## 3. Discussion and conclusions

We have reported a case with clinical manifestations consistent with the syndrome of FOSMN first described in 2006 by Vucic and his colleagues.^[2]^ One hundred cases of FOSMN syndrome have been reported to date.^[3]^ FOSMN syndrome is typically characterized by the onset of facial paresthesia and sensory deficits in the facial trigeminal innervation area, with slow development along the rostral-caudal direction, affecting the scalp, neck, upper trunk, and upper limbs in a gradually progressive manner.^[2–5]^ At the same time or later than the sensory symptoms, the cranial nerve and upper limb motor disorders also appear slowly, spreading in the same pattern as the sensory disorders. Clinical symptoms and signs include asymmetric facial paralysis, masseter and temporal muscle weakness, dysarthria, dysphagia, tongue atrophy, and even dyspnea in a few patients, which may eventually lead to respiratory failure.

In our case, the patient had typical clinical manifestations, that is, the trigeminal sensory symptoms are followed by lower motor neuron disease with bulbar onset. Blink reflexes displayed bilateral delayed R1 and R2 responses, FOSMN is diagnosed. However, the fluctuation of the patient symptoms, decreasing amplitude of low frequency repetitive electrical stimulation, positive anti-AchR antibody and anti-titin antibody, which were unrelated to FOSMN syndrome.

Moreover, improvement of the patient symptoms after treatment with pyridostigmine, we considered the diagnosis of MG. But in terms of pathogenesis, FOSMN and MG are 2 completely different diseases. MG is an autoimmune disorder of neuromuscular junction transmission disorders, and the lesion site is in the postsynaptic membrane of the neuromuscular junction, unlike many autoimmune diseases, MG autoantibodies are demonstrably pathogenic.^[6]^ So far, the nosogenesis of FOSMN have not yet been fully elucidated, the primary speculations are neurodegeneration and autoimmunity. The common pathological features of FOSMN autopsy showed the loss of specific neurons selectively involved in the brainstem and spinal cord, including the loss of motor neurons in the facial nerve nucleus and hypoglossal nucleus and anterior cervical horn, the loss of sensory neurons in the trigeminal main sensory nucleus and solitary nucleus, and the dorsal root ganglion. TAR DNA-binding protein 43 positive inclusion bodies were found in glial cells in several patients.^[2,7,8]^ The abovementioned pathological research evidence supports that FOSMN syndrome is a neurodegenerative disorder and a likely amyotrophic lateral sclerosis association with amyotrophic lateral sclerosis.^[7–10]^ De Boer reviewed 100 FOSMN patients and discovered that Autoantibodies were positive in 14 of them, and that immunotherapy is partly effective in these patients,^[3]^ however without any cases of anti-AchR antibody and anti-titin antibody. These discoveries indicate the probability of a potential immunological mechanism. Therefore, further fundamental studies and clinical reports are needed to certify the relationship between the 2.

In summary, FOSMN syndrome and MG have distinct clinical features. Herein, we reported a rare case in which both conditions co-existed. This case suggests that FOSMN syndrome maybe, in part, immune mediated. Our case report might expand the spectrum of FOSMN.

## Acknowledgements

All authora would like to acknowledge the patient and her family for generously permitting the use of the data in this report.

## Author contributions

**Data curation:** Chengyu Pan, Zhiwei Zhou.

**Formal analysis:** Zhenzhen Tai, Renfang Hao, Jin Wang.

**Writing – original draft:** Chengyu Pan, Xiangrong yang.

**Writing – review & editing:** Tao Liang.

## Supplementary Material


